# The role of infection prevention and control in the mitigation of human-to-human transmission of Nipah virus: a systematic review

**DOI:** 10.1186/s13756-025-01677-5

**Published:** 2025-11-29

**Authors:** Samuel Pritchard, Emilio Hornsey

**Affiliations:** 1 London School of Hygiene and Tropical Medicine, London, UK; 2 UK Public Health Rapid Support Team, London, UK

**Keywords:** Nipah, Infection prevention, Human transmission, Nosocomial, Systematic review

## Abstract

**Background:**

Detected first in humans in 1998, Nipah virus (NiV) has produced regular outbreaks with high mortality rates in Southeast Asia. Outbreaks are often driven by human-to-human transmission, posing an infection risk to both the wider community and healthcare workers who care for NiV cases. Infection prevention and control (IPC) practices are key to preventing amplification of disease outbreaks and protecting both healthcare workers and other patients, but significant evidence gaps exist in the identification of specific modes of NiV transmission that occur in outbreaks and the effectiveness of currently recommended IPC practices against NiV.

**Methods:**

A literature search was conducted, retrieving studies from PubMed, Embase, and medRxiv. Following screening, 32 eligible studies were included in the review. Selected studies described the modes of transmission identified in NiV outbreaks, effectiveness of IPC practices against NiV and specific risk factors for NiV transmission. A meta-analysis of NiV transmission exposure risk factors was subsequently conducted.

**Results:**

NiV transmission was identified following various types of contact with cases, including direct contact, touching body fluids and contact with a case after death. A minority of cases were responsible for most transmission events, with 94 cases (67%) with identified transmission routes generated from 6 cases (4%). No comparative studies were found that assessed IPC effectiveness, but several studies described examples of shortcomings in IPC provision in NiV outbreaks. Meta-analysis identified contact with a NiV case as a significant risk factor for transmission.

**Conclusions:**

Identification of multiple modes of human-to-human transmission stresses the importance of adherence to recommended IPC practices when caring for NiV patients. Several nosocomial outbreaks coincided with deficiencies in the wearing of personal protective equipment, hand hygiene, and surface cleanliness. The successful application of updated global IPC recommendations would represent a significant advancement of global preparedness for NiV outbreaks to prevent disease transmission and reduce the risk of a NiV epidemic or pandemic.

**Supplementary Information:**

The online version contains supplementary material available at 10.1186/s13756-025-01677-5.

## Background

Nipah virus (NiV) is an emerging zoonotic *Henipavirus* (family *Paramyxoviridae*) that is enzootic among *Pteropus* bats in Southeast Asia [1] and causes sporadic outbreaks of severe disease in humans. Two distinct NiV genetic clades have been identified, with NiV-M responsible for the initial outbreak in Malaysia and NiV-B causing regular outbreaks in Bangladesh and India [2]. Human infection typically occurs between 4 and 18 days following exposure to NiV [3]. The estimated case fatality rate (CFR) in human outbreaks is 40–75% [4], with some outbreaks in Bangladesh exhibiting CFRs of over 90% [5].

Since its emergence in the human population in 1998, over 600 cases of NiV have been recorded globally [6]. NiV outbreaks emerge following zoonotic transmission to humans, which occurs either via contact with fruit bats, consumption of contaminated raw date palm sap [7] or eating fruits half-eaten by an infected bat. The first recorded NiV outbreak began in Malaysia in September 1998, which predominantly impacted pig farmers and abattoir workers [8], implicating pig-to-human transmission as the primary driver of the outbreak. Since Bangladesh’s first outbreak in 2001, NiV has demonstrated human-to-human transmission in Bangladesh and India [9, 10], with many outbreaks taking place within healthcare facilities. Multiple retrospective analyses of NiV cases in Bangladesh have suggested that contact with a NiV case is a significant risk factor for infection [11, 12]. Human-to-human transmission is hypothesised to occur either via direct contact with infected cases or contact with infected body fluids, such as saliva or urine [13]. The extent to which NiV outbreaks have been propagated by human-to-human transmission varies significantly, with some outbreaks primarily driven by date palm sap consumption with limited human-to-human spread [14]. The R_0_ for NiV, based on 14 years of outbreak data in Bangladesh, was estimated at 0.33 (95% confidence interval: 0.19–0.59) [15], indicating that the current probability of a large NiV outbreak, given available information, is rare.

Within a global environment fertile for zoonotic infectious disease emergence, NiV’s high case fatality rate, ability to transmit between humans, and the absence of effective medical countermeasures render it a significant potential threat to global health security. Anxiety regarding the threat to global health posed by NiV is reflected by its classification as a Biosafety Level 4 (BSL-4) pathogen and its designation by the World Health Organization (WHO) as a priority pathogen for research and development (R&D) in a public health emergency context. A WHO ‘Pathogens Prioritization’ report, published in July 2024, updated the organisation’s R&D blueprint and outlines paramyxoviruses as ‘high’ risk of causing an epidemic or pandemic [16].

Central to the global recognition of NiV as a major public health concern is the observation of human-to-human transmission. Several outbreaks have indicated an increased risk of infection in those with history of contact with a NiV case [17], including healthcare workers (HCWs): a key barometer for human-to-human transmission of any pathogen. The high virulence of NiV disease means infected individuals invariably require significant contact with healthcare teams during the infectious period [18], rendering HCWs a particular risk group during a NiV outbreak. An outbreak of NiV among HCWs, due to the high CFR, could be catastrophic to the provision of healthcare in an outbreak region. As a result, it is imperative that the optimum strategies to prevent nosocomial transmission of NiV are implemented, which this project attempts to identify.

At the time of writing, no licensed drugs or vaccines exist for NiV treatment or prevention, hence epidemiological methods are relied upon to control NiV outbreaks. Case identification and isolation plays a central role in the response to a NiV outbreak, while WHO and various national ministries of health recommend the use of IPC precautions to protect HCWs from infection (CITE).

The ability of NiV to transmit between humans, an absence of effective drugs or vaccines, and the inconsistent availability and use of PPE in regions prone to NiV outbreaks, hinders the care of NiV patients and threaten to amplify outbreaks, potentially to the level of a major international health crisis. Identifying the specific modes of transmission, and the IPC precautions most efficacious in interrupting transmission, should help guide epidemic preparedness and inform response in nations affected by NiV outbreaks to minimise transmission risk, especially within healthcare settings. This review aims to elicit a greater understanding of the specific modes of transmission of NiV, as well as inform the development of IPC guidelines for NiV to protect HCWs that may encounter cases of the disease in their practice.

## Methods

### Development of search protocol

A literature search was carried out using Ovid, searching PubMed, Embase and medRxiv databases for articles published prior to 1 June 2024. Three search questions were developed based on the objectives outlined in the Aims and Objectives section; these are outlined below. The full search protocol is outlined in Supplementary File A.

1. What are the modes of human-to-human transmission of NiV?
2. Which IPC precautions are most effective in preventing transmission of NiV to healthcare workers?
3. What are the risk factors for transmission of NiV within healthcare-based or community settings?

The population, intervention, comparison and outcome (PICO) question format was chosen for questions 2 and 3 in this review. Question 1 did not require the PICO format as the question is inherently descriptive, and therefore choosing intervention and comparisons would not be suitable. Three IPC precautions were chosen as interventions for question 2: use of an airborne precaution room, wearing an N95 respirator and the use of hand hygiene among HCWs. These precautions were chosen to align with the methodology of a WHO rapid review on human-to-human transmission of mpox, as well as their relevance to existing national and subnational guidelines on IPC for NiV [19, 20]. Isolation of confirmed and probable cases, whether at home or while receiving treatment at a facility, was chosen as the intervention outcome for question 3, which was compared to cases that were not isolated during their infectious period.

Geographical limitations were applied to the search protocol. As NiV outbreaks have only occurred in Bangladesh, India, Malaysia, Philippines and Singapore, only studies from these five countries were included in the search. Limits were placed on all questions with respect to year of publication; NiV was discovered in 1998, so papers published prior to 1 January 1998 were excluded from the search, along with articles published in languages other than English.

### Literature screening

**Fig. 1:**
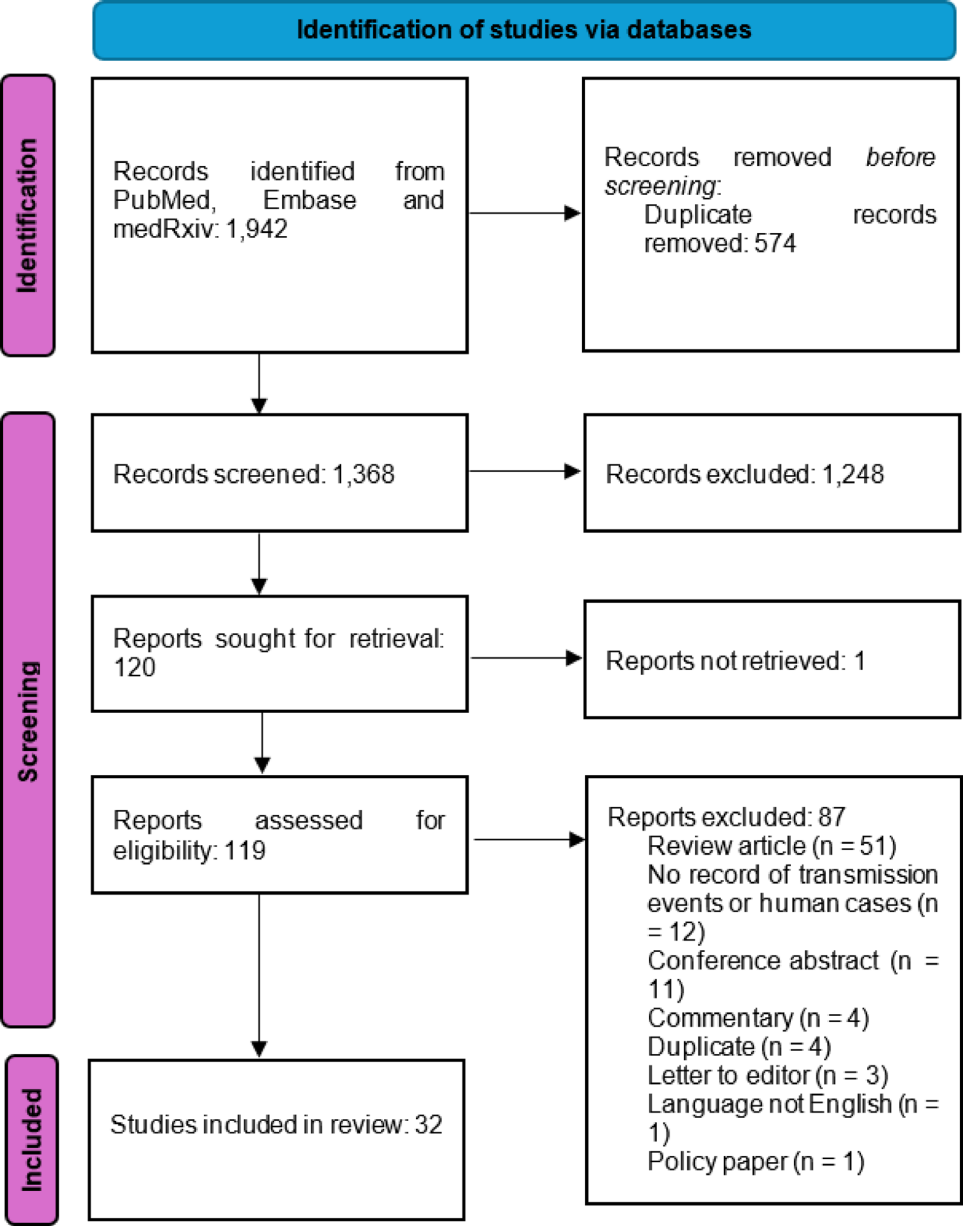
PRISMA diagram outlining the number of results generated by the literature search and subsequent screening

The literature search produced 1,942 initial results. Following deduplication, title and abstract screening was conducted on 1,368 records. Papers excluded at this stage included studies focused solely on the structural or molecular biology of NiV with no record of transmission events or human cases of NiV (n = 350), studies focused on developing medical countermeasures for NiV (n = 203), studies focusing on pathogens other than NiV (n = 153), animal studies (n = 110) and studies evaluating zoonotic rather than human-to-human transmission of NiV (n = 86). 120 papers were therefore eligible for full text screening, of which one paper could not be obtained, with full text screening leaving 32 eligible studies. Figure 1 shows the PRISMA diagram describing the literature search.

### Quality and risk of bias assessment

Papers that met the inclusion criteria for the search were subsequently assessed for quality and risk of bias. For comparative studies, this was conducted by adapting the JBI Critical Appraisal tools for case–control and cohort studies to form a checklist applicable to all eligible comparative studies in this review. Descriptive studies, such as outbreak reports and case series, were assessed using the JBI checklist for case series. Eligible papers were sorted into comparative and descriptive studies prior to assessment. Questions in each checklist were answered with ‘yes’, ‘no’, ‘mixed’, ‘unclear’ or ‘n/a’ for each paper. The checklists used in this assessment and the results for each included study are listed in Supplementary File B.

### Data extraction and analysis

Papers accepted following quality and risk of bias assessment qualified for data extraction. Three different data extraction tables were prepared, with one for each review question. The time of study, location and study subject population were recorded for papers in all data extraction tables. For all questions data from Malaysia, the Philippines and Singapore were analysed separately to those from Bangladesh and India, owing to the epidemiological differences observed in NiV outbreaks between these countries [6].

For question 1, descriptive case data were recorded, including dates for exposure, illness onset, hospitalisation and death, as well as history of exposure to hospitals, other NiV cases and environmental risk factors. Overall estimates were calculated for the incubation period, symptomatic period in fatal cases and case fatality rate. The incubation and symptomatic period calculations and distribution were provided to aid in identification of time windows for contact tracing, assessment of whether pre-symptomatic or asymptomatic transmission may have taken place, and facilitates understanding of timing for quarantine of contacts. The proportions of cases arising from human-to-human, nosocomial, community and zoonotic transmission were also recorded, and transmission chains mapped where identified. Human-to-human transmission was defined as the development of NiV in a person who had reported contact with a confirmed or probable NiV case between 4 and 18 days prior to illness. If a case had history of exposure to both human and zoonotic sources of NiV, comparative data from case–control studies from the outbreak in question were used to inform the classification of whether specific NiV transmission events occurred as human or zoonotic transmission. Following this assessment, if a case’s exposure history remained ambiguous, transmission would be defined as having an unidentified source. If contact took place in a healthcare setting, the transmission was classed as nosocomial, with all other recorded human-to-human contact classed as community transmission. Human-to-human transmission was broken down into proportions by specified route of contact, such as droplet or aerosol transmission and body fluid contact, where data were available.

Comparative studies identified for questions 2 and 3 had their effect size and 95% confidence interval recorded for the efficacy of IPC precautions and risk factors for NiV infection respectively. For outcomes where sufficient data were available, a fixed-effect meta-analysis was conducted to evaluate the association between different IPC precautions and risk factors with NiV infection. Meta-analysis weighting was based on the inverse variance of each study: to calculate this, standard errors were retrospectively calculated from confidence intervals of the effect sizes from each study. Descriptive data about the use of IPC precautions were also collected where available to provide case studies of the variable implementation of precautions during NiV outbreaks.

## Results

### Description of included studies

Overall, 32 studies met the inclusion criteria for the review and were accepted for data extraction following the quality and risk of bias assessment. The breakdown of the review questions addressed by each study is outlined in Supplementary File C. Some studies contained information relevant to multiple questions: for example, outbreak reports that contained both an exposure history for each NiV case as well as a case–control study for specific risk factors for infection were included in the analyses for questions 1 and 3. Of the 29 studies describing and analysing NiV outbreaks, 15 were conducted in Bangladesh, 8 in India, 4 in Malaysia and 2 in Singapore, spanning the years 1999–2023.

### Modes of human-to-human transmission of NiV

In Malaysia, the Philippines and Singapore, 2 studies gave descriptive accounts of NiV outbreaks, with 1 from Malaysia and 1 from Singapore, from which no documented human-to-human transmission was identified [21]. Serological studies in both countries corroborated this hypothesis, with no serological evidence of previous NiV infection found among 591 HCWs across Malaysia and Singapore with a history of exposure to NiV patients [22, 23]. As the transmission of NiV in these three countries is primarily driven by pig-to-human contact, in-depth assessment of human-to-human transmission networks was not possible.

11 of the 23 studies conducted in Bangladesh and India, covering 10 separate outbreaks, provided descriptive information regarding the exposure history of NiV cases within outbreaks. In total, studies documented 189 cases of NiV occurring between 2001 and 2023: 113 (60%) were male and 76 (40%) were female. The median age among the cases could not be identified, as most studies did not provide individualised age data. 148 cases (78%) had sufficient information to infer the source of transmission, with 133 linked to exposure to a human NiV case and 15 linked to zoonotic exposure, as described in Table 1.

**Table 1:** Source of NiV infection in identified outbreaks in Bangladesh and India, 2001–2023

Source of NiV transmission	Number of cases (% of total)
Human NiV case	133/189 (70%)
Zoonotic	15/189 (8%)
Unidentified	41/189 (22%)

**Fig. 2:**
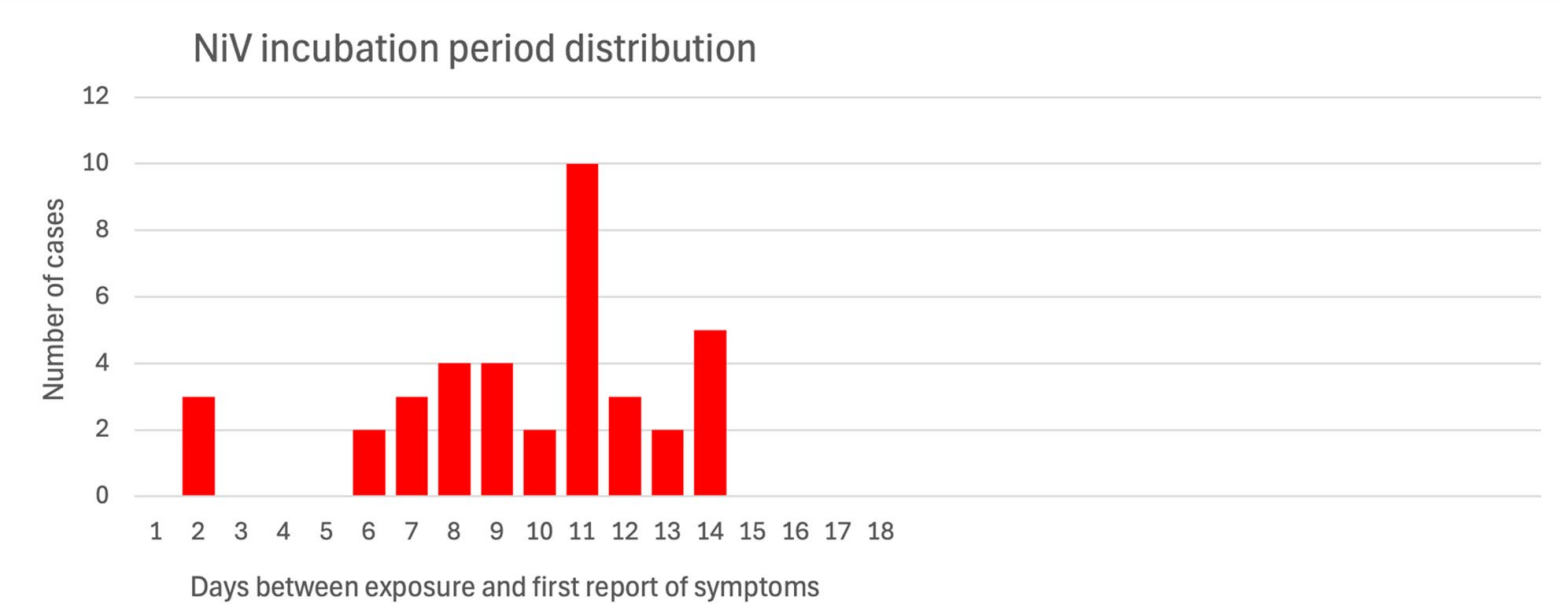
Distribution of the length of incubation period for 38 NiV cases where data for 1) date of exposure and 2) date of onset of symptoms were available. For cases with multiple exposures across different dates, the date of first exposure was used in the analysis

From the 38 cases where dates of exposure and illness onset were identified, the mean incubation period was 9.71 days (minimum: 2 days, maximum: 14 days, IQR = 8–12 days). The mean symptomatic period was 5.47 days (minimum: 0 days, maximum: 13 days, IQR = 4–7 days); this was calculated from the 36 cases that had recorded dates of illness onset and death. No information was available regarding the date of symptom resolution in non-fatal cases. Incubation period data is shown in Fig. 2.

**Fig. 3:**
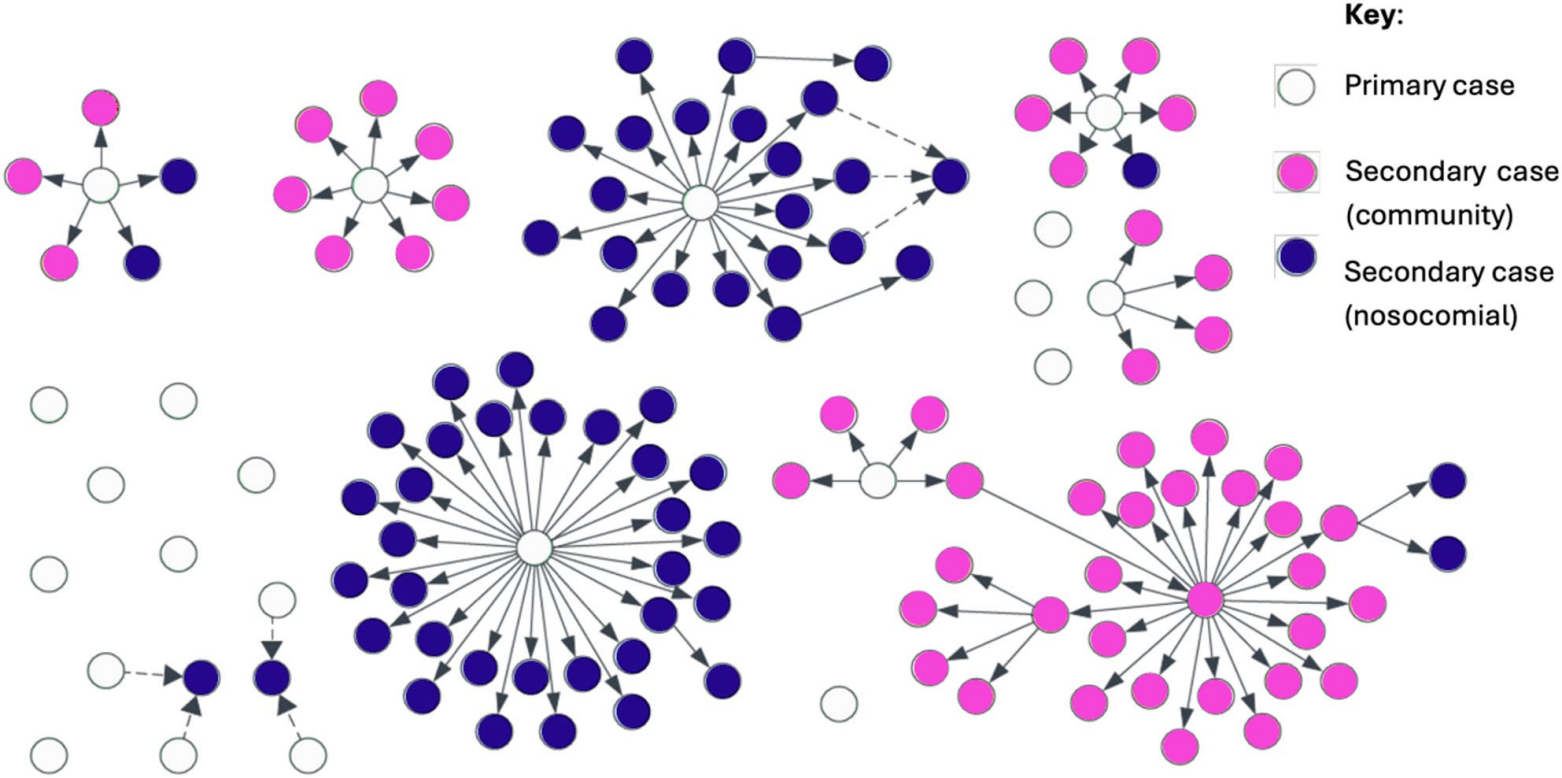
Visualisation of NiV transmission clusters in India and Bangladesh, 2001–2023. Green circles represent primary cases with zoonotic or otherwise unknown origin, orange circles represent cases infected in the community, and blue circles represent cases who were infected in a nosocomial setting. Solid lines represent identified transmission events, with dashed lines used when there 2 or more candidate primary cases were suspected for a transmission event

Of the 133 cases that were acquired from human contact with a NiV case, 61 (46%) were acquired via nosocomial transmission, with 72 (54%) occurring following transmission within the community. 36 cases (27%) were family members of another case: these cases arose following contact in both nosocomial and community settings. There was significant heterogeneity in terms of the number of secondary cases generated by NiV cases; among 133 cases acquired from human contact with a case, 94 (71%) were generated from transmission from just 6 primary cases, with 9 instances of cases producing 4 or more secondary cases. Events where a case transmitted to multiple contacts were observed in both healthcare settings and the community. Figure 3 maps the transmission of all 131 cases where both the specific transmission origin (i.e. either a zoonotic source or a specific individual from whom said cases acquired infection) and direction of transmission are known [10, 17, 24–28].

**Fig. 4:**
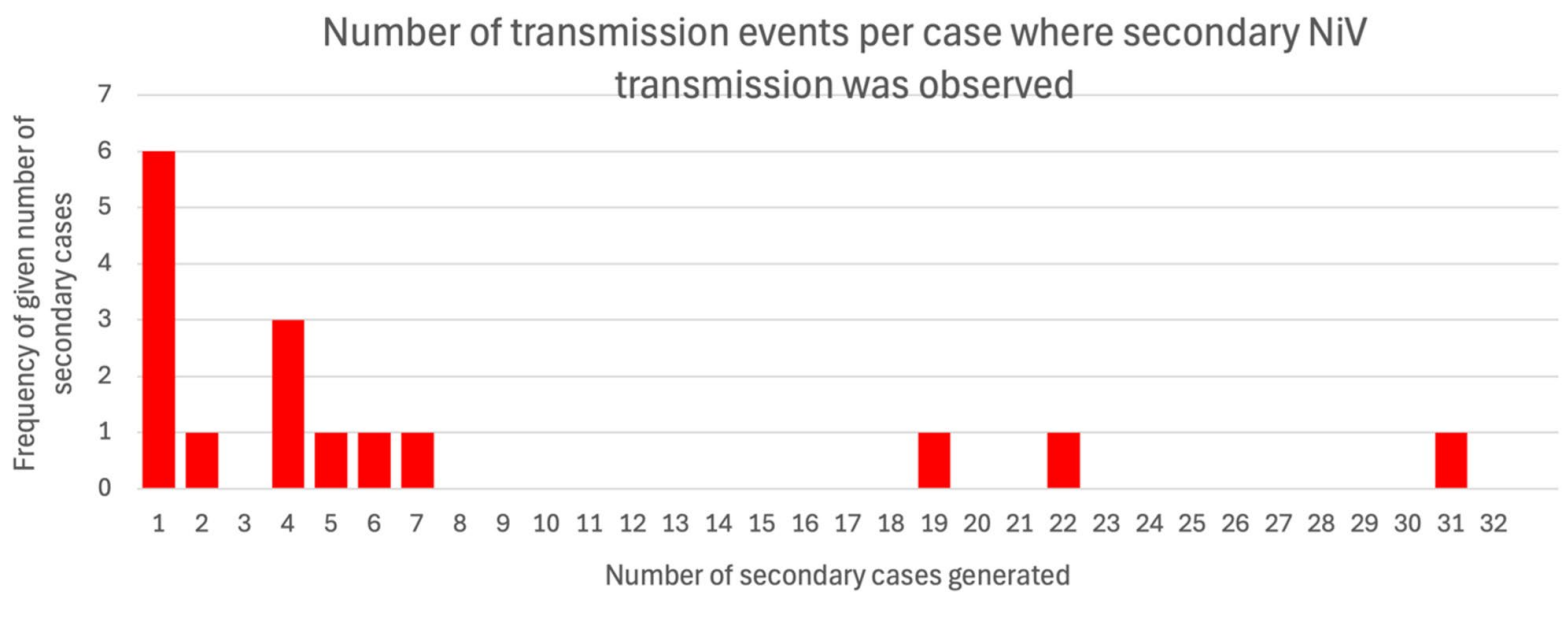
Distribution of the number of secondary NiV cases generated by each case among the 16 cases of NiV identified in the review that produced secondary transmission. For interpretative purposes, the chart excludes the 115 NiV cases which did not generate secondary cases, allowing easier visualisation of examples of where human-to-human transmission took place

**Fig. 5:**
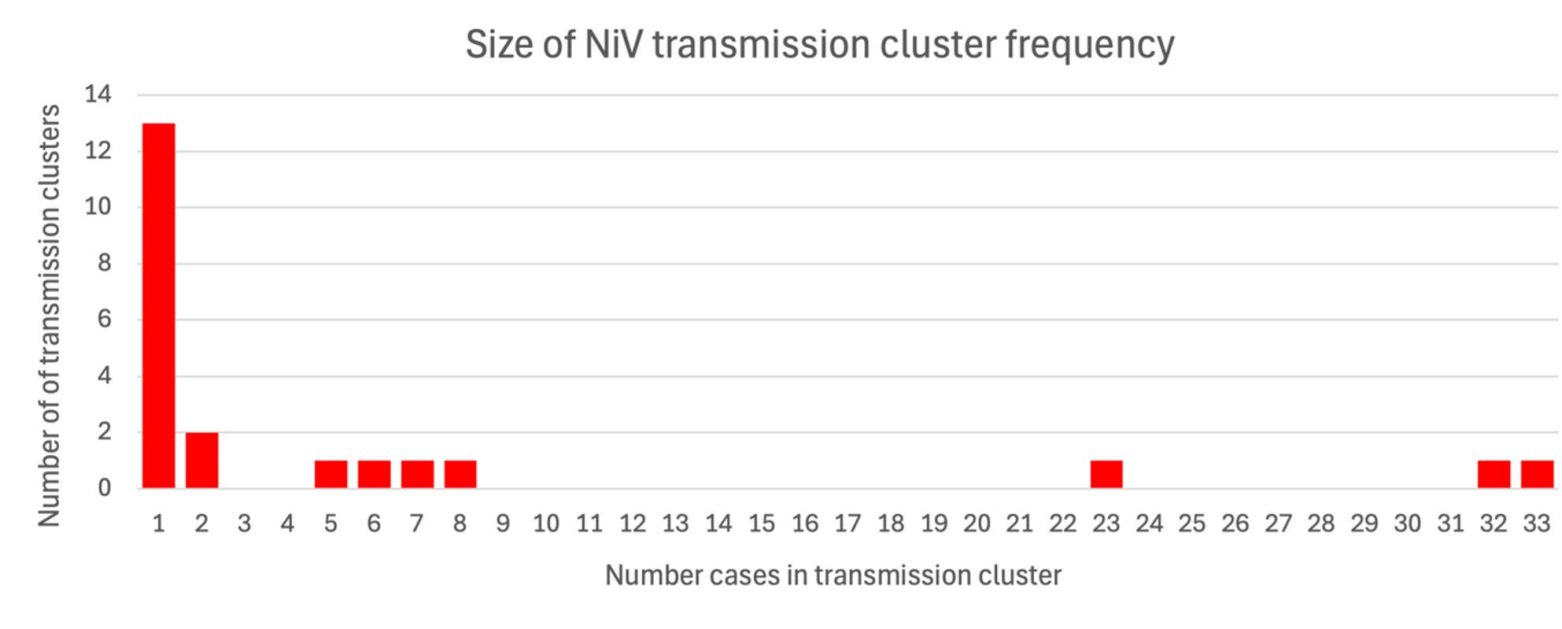
Distribution of the size of 22 NiV transmission clusters identified within descriptive accounts of NiV outbreaks. Out of the 22 clusters reviewed, 13 (59%) involved a single case, indicating that the majority of introductions of NiV to humans identified in this review did not produce secondary transmission

The analysis of transmission clusters included inference of average transmission chain length, which was 1.43 transmission events per chain. There were 6 instances of fourth-generation transmission of NiV identified in the analysis. The figure for average transmission chain length is likely an overestimate of the true figure for NiV, as studies documenting single-case NiV outbreaks were excluded from the review. The mean number of secondary cases generated by each case was 0.83, with 5.95 being the average number of cases per transmission cluster, which is further illustrated in Figs. 4 and 5 respectively.

Data on the types of human-to-human contact that led to NiV transmission were difficult to obtain, as a significant number of cases had history of multiple types of contact with another case prior to developing NiV infection. Of the 46 cases where case exposure histories included one or more route(s) of contact, 17 (37%) had direct physical contact with a case during their illness, 8 (17%) had contact with a case’s body fluids, 35 (76%) were present in the same room as a case at any given time, and 3 (6%) had direct contact with a case after their death. Three studies also described contact via sharing of food and sleeping in the same bed as a case, which was reported as a route of contact by 1 and 5 cases respectfully [17, 26, 27], but it could not be confirmed that transmission occurred via these routes due to other types of contact having also taken place alongside them. The descriptive literature provided no evidence of vertical transmission of NiV in pregnancy or sexual transmission.

### Meta-analysis of risk factors for healthcare-based and community NiV transmission

The literature search identified 12 comparative studies analysing potential risk factors for NiV infection, of which 11 were case–control studies. Of these, 7 studies included contact with another NiV case as an exposure. Odds ratios (ORs) could not be calculated in 2 out of 7 studies, as both studies had either an exposed or unexposed cohort that had zero cases. As a result, 5 studies providing ORs for the development of NiV infection following contact with a NiV case were eligible for meta-analysis. The low case numbers associated with NiV outbreaks meant that most case–control studies from individual outbreaks had low power and it was often difficult to provide definitive conclusions for the risk factors for NiV infection.

**Fig. 6:**
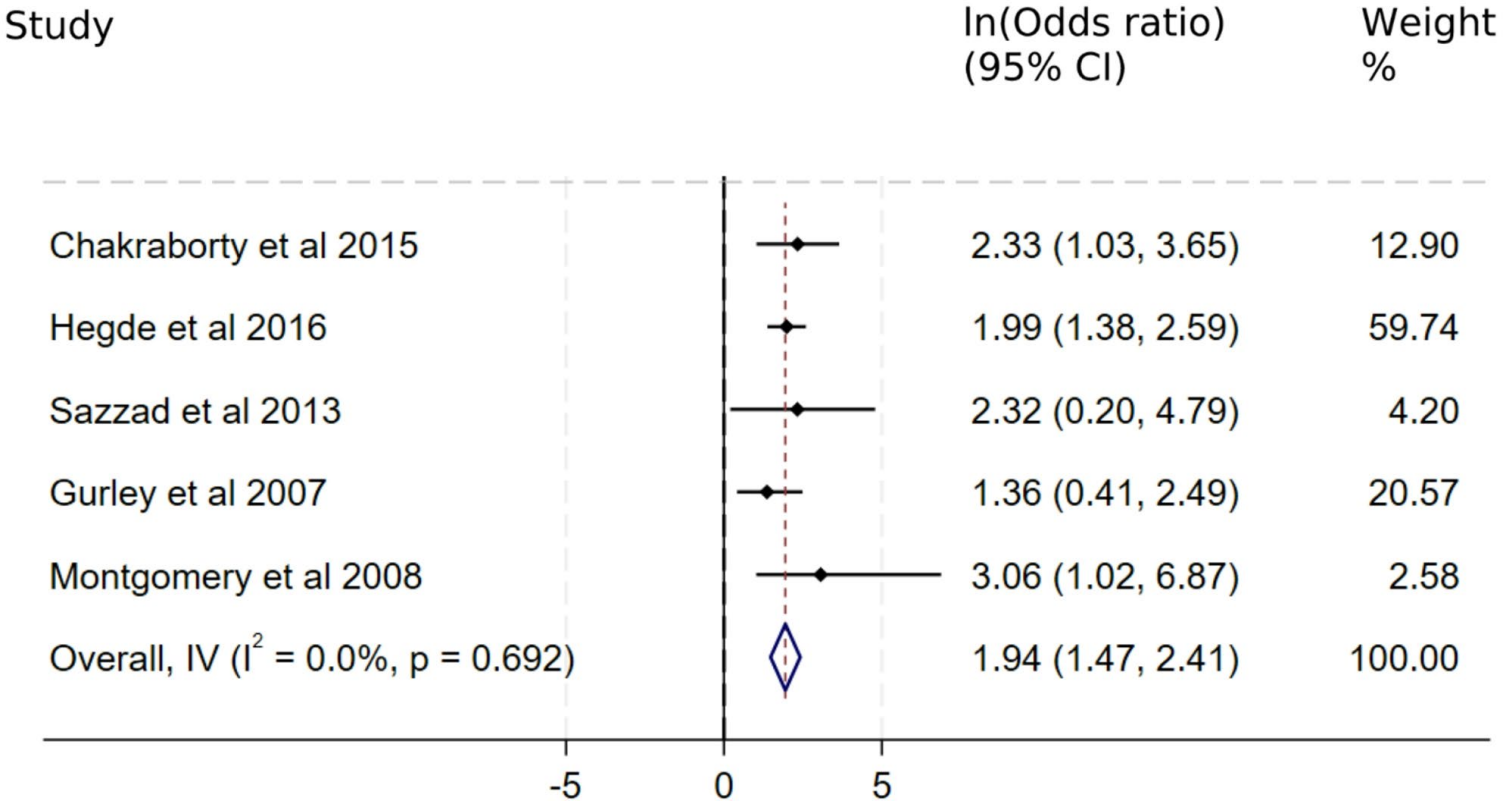
Meta-analysis of studies measuring odds of NiV infection in those who had contact with NiV cases. Odds ratios are presented in natural logarithms for ease of interpretation

The 5 studies include in the meta-analysis were conducted in Bangladesh [11, 12, 26, 28, 29]. No case–control studies from India assessed the odds of NiV infection following contact with a case. Contact with a NiV case was significantly associated with acquiring NiV infection in all eligible studies. The natural logarithm of the OR produced by the meta-analysis for the 5 studies was 1.94 (95% confidence interval: 1.47–2.41), translating to an OR of 6.99 (4.37–11.18). The results of this meta-analysis are presented in Fig. 6.

There were limited case–control study data from only 1 study that measured contact with another NiV case via specific transmission routes. A study from a 2001 outbreak in Meherpur, Bangladesh identified caring for or living with a case (OR: 4.80, 1.23–18.8) and contact with a case’s body fluids (5.73, 1.00–32.7) as potential risk factors, with no increased risk from sharing personal items (0.49, 0.09–2.56). 3 studies analysed the risk of being in the same room as a NiV case, with inconclusive results: ORs of 0.59 (0.08–3.2) and 2.17 (0.77–7.43), with one study not able to calculate an OR. Visiting a hospital or clinic was measured as a risk factor in 2 studies, which both indicated an increased risk of NiV infection in visitors, with ORs of 4.45 (2.53–7.84) and 32.4 (5.18-inf). However, both studies carried a significant risk of confounding, as they did not control for the hospital visits of NiV cases requiring medical care.

### Impact of IPC precautions on NiV transmission

The literature search provided no comparative studies on the use of any specific IPC precautions in the prevention of NiV infection in healthcare workers. As a result, systematic review of the use of IPC in preventing NiV transmission was not possible in this project, and answering this question relied on collating descriptive information from outbreak reports and other studies mentioning the use of IPC, as opposed to directly evaluating which IPC measures would be effective in a future NiV outbreak.

There were several described examples from the literature of NiV outbreaks where IPC practices were deemed insufficient. Analysis of a 2001 outbreak in Siliguri, India, identified gaps in IPC practices prior to the outbreak, in which 45 of 60 cases arose from nosocomial transmission. The use of medical masks and respirators was limited outside of the intensive care unit (ICU), wards were often overcrowded with patients and there was no compliance with IPC protocols in the cleaning of surfaces and laundering of bed linen [24]. A 2018 outbreak in Kerala, India, where all 22 identified transmission events were nosocomial, noted that no further transmission occurred following isolation of cases in a separate ward and initiation of stricter barrier nursing protocols [30]. Prior to this, the two hospitals where transmission occurred were overcrowded and not ventilated properly, with hospital staff not wearing PPE or performing hand hygiene [31]. Similar protocols were enforced following detection of another Kerala outbreak in 2023, with no cases detected following implementation [25].

Several studies described the current inadequacy of IPC practices in Bangladesh [12, 32, 33], describing insufficient supplies of PPE, lack of available handwashing stations and the reliance on family members to act as caregivers in hospitals. A 2010 outbreak in Faridpur District, Bangladesh, documented a physician in training who died of NiV infection following examination of 5 NiV cases without washing his hands or using a mask or gloves [26]. An analysis of two NiV outbreaks in 2001 and 2003 showed that although no HCW infections were detected, only 40% of HCWs used gloves, masks or gowns when treating patients. The analysis also identified a needlestick injury and mucous membrane contact with body fluids from a NiV case among HCWs, although NiV transmission was not observed following these instances [34]. Another study reported that only 28% of hospital workers, including 19% of physicians, wore PPE “most of the time”, with limited use of masks, gloves and eye protection [35].

A 2019 cross-sectional analysis of adherence to National Airborne Infection Control (NAIC) guidelines across 50 hospitals in Kerala provided quantitative insight into IPC practices within the region. Contrary to NAIC guidelines, only 5 (10%) of all hospitals in the analysis used N95 respirators during contact with patients with respiratory symptoms, and only 10 (20%) hospitals isolating patients with respiratory symptoms. Governance of IPC showed similarly inconsistent results, with only 20 hospitals aware of the national Ministry of Health and Family Welfare (MHFW) guidelines for IPC, and 9 (18%) hospitals, of which none were public hospitals, conducting risk assessments for airborne pathogens [36].

## Discussion

70% of NiV cases identified in India and Bangladesh reviewed in studies included in the review arose from human-to-human transmission, which is consistent with the conclusions of other retrospective analyses of multiple years’ worth of NiV cases [15, 17]. The incubation period calculated from identified cases was also in line with estimates from other reviews [3, 37]. In this analysis, NiV transmission in these countries was characterised by short transmission chains and sporadic ‘superspreading’ events; out of 133 cases generated from human-to-human transmission, 94 (71%) were generated from just 6 cases. Three NiV cases each produced 19 or more secondary cases [24, 28, 31], two of which occurred in hospitals following admission of a NiV case. Human-to-human transmission was identified in both healthcare and community settings, with contact with another NiV case representing a major risk factor for infection. Other potential risk factors where available evidence was either minimal or mixed included being in the same room as a NiV case, caring for a case, and contact with body fluids.

Proposed human-to-human transmission routes for NiV included direct contact with a NiV case [10, 17, 26–28], their body fluids [10, 17, 26, 27], or their body following death [26]. 18 NiV cases were also identified in individuals who recalled being in the same room as a case but had no recorded history of contact with a case via these routes [10, 17, 31], which suggests that fomite transmission and/or transmission via the air may have also contributed to outbreaks. Some cases reported a history of contact with oronasal secretions [26, 27, 31] and vomit [17]. There was no evidence of infection following contact with blood, semen or other body fluids, but this does not exclude them as potential sources of transmission. Case exposure histories were not consistently provided or necessarily complete in descriptive accounts of NiV outbreaks, making it difficult at times to identify the nature of contact with cases, let alone isolate the type of contact that was responsible for transmission. The finding of multiple routes of human-to-human transmission of NiV underlines the urgent need for comprehensive implementation of an IPC strategy that targets all of these routes.

There was significant variation in the number of secondary cases generated from each case, with three instances of infected individuals infecting 19 or more others [24, 28, 31]. One household cluster in Bangladesh in 2004 demonstrated a secondary attack rate of 80% [28]. Analysis of the transmission clusters in this review showed that most transmission events occurred as part of superspreading events. This suggests that significant progress can be made on control of NiV outbreaks by developing strategies to identify individuals and settings likely to be part of a superspreading event. Two of the three events where one NiV case infected 19 or more others took place in hospitals, both of which involved transmission from the hospital’s first case of NiV. Potential reasons for the magnitude of transmission cited from these outbreaks included inadequacies in barrier control methods and hand washing, long waiting times for investigations and procedures, the regular visits of family and their involvement in patient care [10, 24]. Future research should attempt to characterise the drivers of these events by conducting studies that compare viral shedding patterns between non-transmitters and those generating several secondary cases, as well as characterising the relationship between delays in diagnosing cases of NiV and the degree of human-to-human transmission observed.

Laboratory studies on NiV shedding could also be combined with descriptive accounts of NiV transmission to inform IPC practice: NiV has been previously isolated from respiratory droplets from cases [13], with non-human primate infection documented following exposure to NiV aerosols under experimental conditions [38, 39]. A 2024 review showed significant differences in NiV shedding via the respiratory route among infected African green monkeys [6], which, if replicated in humans, could provide one explanation to the huge variation in the number of secondary cases generated by each NiV case. A study of NiV aerosol persistence concluded that NiV particles can remain in the air for up to 90 min at 20 degrees Celsius [40]. Detailed description of case exposures that specifically rule out other types of direct and indirect contact, other than being in the same room as a NiV case, may provide further backing to the hypothesis of NiV transmission via the air, but case–control studies that analyse this as a risk factor showed varied results for different outbreaks [14, 27, 28].

Likewise, the descriptive literature provided little explicit evidence for fomite transmission of NiV, but the emergence of cases without history of direct contact with another case mean it cannot be ruled out. A 2018 nosocomial outbreak in Kerala produced 22 secondary cases, most of which did not have documented history of direct contact but were in the same room as a NiV case [10, 31]. Studies that isolate NiV from hospital surfaces and analyse survival may fortify the hypothesis that NiV can be transmitted through surfaces. This would necessitate more stringent cleaning practices in hospitals: these were found to insufficient in some NiV outbreaks, where surfaces were left visibly soiled and contaminated by infected body fluids [28, 33].

While there was a lack of comparative study data on the efficacy of specific IPC measures against NiV transmission, the transmission routes identified in the descriptive literature provide some basis on which to make recommendations for IPC measures. Transmission-based precautions, such as gloves, respirators, aprons and eye protection, provide significant protection against pathogens transmitted via direct and indirect contact routes [41], while improving ventilation has been shown to reduce aerosol persistence in indoor air [42]. Studies that aim to identify high-risk medical procedures, which could represent case–control studies for the risk of NiV infection following specific procedures, should be prioritised in future research efforts.

The swift implementation of case isolation and enhanced IPC measures in affected hospitals coincided with rapid control of outbreaks [25, 31]. This practice could be enhanced by comprehensive implementation of standard precautions, early screening for infectious symptoms and use of transmission-based precautions, which was exposed as inadequate within Kerala hospitals in 2019 [36]. Development and deployment of rapid diagnostic tools and syndromic surveillance would mean index NiV cases are identified and isolated earlier, reducing opportunities for nosocomial transmission. Index cases, even in 2023, are often not confirmed until after their death [25, 31], providing no opportunity for IPC measures initiated at outbreak detection to have any impact during the illness of the index case.

The finding of transmission to individuals encountering NiV cases after their death presents a unique challenge regarding IPC. This review showed that funeral and burial practices in Bangladesh present opportunities for contact with body fluids [26]. The high case fatality rate of NiV means a substantial proportion of cases will die from their illness in a healthcare setting, where transportation of the body and post-mortem examination may be carried out: any NiV-specific recommendations for IPC practice should reflect the risks associated with these procedures. Other HCWs who are not directly involved in patient care should also not be overlooked in IPC recommendations: transmission to a hospital bus driver occurred in a 2007 outbreak in Bangladesh [27],

The identification of hospitals as facilitator of NiV transmission provides a warning for future NiV outbreaks. Hospitalised cases and those at a later disease stage demonstrated a higher number of contacts in an assessment of over 1,500 NiV case contacts in Bangladesh [32], whereas a 2019 analysis suggests NiV transmissibility is higher in cases with breathing difficulties and low survival [15]. As a result, those involved in the care of severely ill NiV cases could be at particular risk of infection. This confluence of factors has been displayed in two outbreaks in India, in 2001 and 2018, where each outbreak generated clusters of over 20 secondary cases from just one index case presenting to a hospital [10, 24], one of which infected 23 staff. The consequences of a nosocomial outbreak of NiV are not limited to the amplification of case numbers. Pressures on healthcare staffing numbers may lead to HCWs working through illness, providing further opportunities for transmission, and deaths of HCWs from NiV would further compound these shortcomings and disrupt routine healthcare service provision.

While this review provides an up-to-date outlook on NiV transmission, the characteristics of transmission outlined here should in no way be interpreted as fixed entities. The dynamics of NiV transmission may evolve based on the emergence of new genetic clades, similar to the varied host range and human-to-human transmission capacity observed in NiV-M and NiV-B, or novel variants may emerge within these existing clades that rely on new routes of transmission or increase overall transmissibility. With just over 600 cases of NiV identified since its emergence in 1998 [6], opportunities for adaptation to human hosts have been relatively rare. Outbreaks with higher case numbers driven by human-to-human transmission may expand these evolutionary opportunities, increasing the risk of emergence of genetic variants with altered transmission properties: a process observed this decade with mpox [43] and severe acute respiratory syndrome coronavirus 2 (SARS-CoV-2) [44].

The findings of this review are not just relevant to countries with a history of NiV outbreaks; those with no previous cases should also be cognisant of the dynamics of how NiV transmits between humans. The distribution of *Pteropus* bats that act as the main NiV reservoir hosts among Southeast Asia, Australia and islands in the Indian Ocean [45], alongside identification of neutralising antibodies against NiV in *Eidolon helvum* bats in Nigeria [46], indicate the potential for expansion of NiV’s host and geographic ranges. Countries not inhabited by fruit bats should still be alert to the risk of imported cases of NiV.

Other practices identified that led to transmission included traditional burial practices, such as the cleaning of the body and its orifices [26], and the reliance on family members as caregivers within hospitals without IPC orientation and support [10]. First and foremost, communication concerning IPC measures must be cognisant of local and religious customs and involve communities in discussions as to how best to encourage risk prevention among individuals, otherwise there risks a breakdown of trust between patients and both HCWs and public health in general.

The application of new IPC measures must also come with a recognition that measures may produce negative effects for patients: a qualitative study in New Zealand found MRSA patients were psychologically affected by isolation and “barriers to normal interpersonal relationships” [47]. This may be particularly pertinent to consider in settings where family members are involved in healthcare, where avenues to providing emotional support to patients undergoing isolation might be necessary.

The final consideration for strengthening IPC is the economic cost of providing measures within low and middle-income countries (LMICs) impacted by NiV, such as Bangladesh. Annual government spending per capita on health in Bangladesh is just $37 [48], higher than only 23 other countries in the world [49], demonstrating the fiscal barriers to improving IPC provision. As a result, other sources of funding may be crucial in determining whether IPC recommendations are implemented where they are needed for NiV. It is also essential that funding for IPC precautions does not fall victim to the ‘panic and neglect’ cycle commonly described in global health financing in periods between outbreaks [50], as it is impossible to predict the exact timing and location of future NiV outbreaks.

### Limitations

Data were predominantly obtained from India and Bangladesh, with limited literature from Malaysia, the Philippines and Singapore identified via the search. NiV cases in Malaysia and Singapore coincided with the initial discovery of NiV, so studies concerned with the outbreak may have used terms such as ‘novel paramyxovirus’, ‘suspected Japanese encephalitis’ and other terms that reflected the aetiological uncertainty associated with the outbreak at the time, thus being missed by the literature search, for which the mention of ‘Nipah’ was a required inclusion criterion. A similar limitation may be relevant to the Philippines, as NiV was never officially confirmed as the aetiological agent for its only outbreak in 2014 [51], which was instead referred to as a ‘henipavirus’ outbreak. Studies not published in English were also excluded from the review, a potential barrier to evaluating locally published sources in India and Bangladesh.

No systematic surveillance for NiV was conducted in Bangladesh prior to 2007 [15], indicating that cases may have been missed in the literature from outbreaks pre-2007. Even in outbreaks where cases may not have been missed, the lack of detail in exposure histories in some studies meant inferring transmission clusters was impossible [17, 29, 34]. One study from a 2001 outbreak in Siliguri, India, could not be retrieved, potentially hindering the detail in which transmission in the outbreak could be described.

It is difficult to measure the incubation period accurately if a case is exposed to several sources of NiV transmission prior to development of NiV illness, or if exposure occurred across multiple days. For this review, the date of first contact with a case was chosen as the date of exposure, so the estimated incubation period may be an overestimate.

Due to the high case fatality rate of NiV, proxy respondents are often relied upon to provide information on behalf of cases, such as exposure histories. These can be subject to recall bias, or proxies may not be able to provide full information about a case. Recall bias may also impact the types of contact reported by contacts or their proxies, resulting in over and underestimates of different contact routes. Types of contact that are more recent, lasting an extended period of time (sleeping with a case) or associated with memorable aspects of disease (e.g. exposure to vomiting) may be recalled more easily and therefore overrepresented in published studies. Similarly, there were 8 instances where NiV cases reported exposure to both zoonotic and human sources of NiV, and while case–control data pointed to human sources as the source of transmission in this outbreak, there remains some uncertainty among these cases with regards to how NiV was acquired.

Comparative studies analysing the risk factors for NiV infection rarely examined the risk of specific routes of contact, rather opting to group these into ‘contact with a NiV case’ as a measurable risk factor. The meta-analysis that measured the risk of contact with a NiV case included one study which measured the risk of contact with a person with fever, which was considered relevant as the study was conducted amidst a NiV outbreak where cases and controls were defined by NiV infection history.

## Conclusion

This review demonstrates, albeit based on limited available epidemiological information, that NiV has the capability to transmit between humans, with individual human cases occasionally producing several secondary cases via human-to-human transmission. The sudden surge in research for control strategies and medical countermeasures for Ebola in 2016 following a multi-country epidemic should not act as a template for the global approach to NiV control. While NiV is yet to cause an epidemic of similar scale as Ebola did in 2013–16, it displays similar disease severity, transmission properties and absence of medical countermeasures to those observed with Ebola pre-2013. An update in global IPC recommendations represent a strategy for controlling NiV outbreaks that can be made based on the best available, if not perfect, evidence that is presented in this review. The next challenge would then to be to ensure implementation of recommendations in countries most susceptible to NiV outbreaks, which coincidentally are among those that experience severe financial barriers to adequate healthcare provision. Ensuring IPC recommendations are considerate of, and compatible with, the cultural context of where they are implemented is a vital consideration for preserving and enhancing trust in public health and disease control wherever a NiV outbreak may emerge.

This review suggests several avenues for future research to further outline the transmission dynamics of NiV and inform control measures. First, the assessment of modes of transmission can be helped by conducting case–control studies from NiV outbreaks that probe several types of human exposure to build a greater evidence base for which types of contact are most associated with developing NiV infection. The development of NiV-specific IPC recommendations should also be subject to regular updates based on the availability of more granular data concerning IPC efficacy: incorporating results from controlled trials comparing the use of specific items of PPE will ensure the highest standards of NiV control are constantly pursued in hospitals.

## Supplementary Information

Below is the link to the electronic supplementary material.

Supplementary Material 1

## Data Availability

The datasets used and/or analysed during the current study are available from the corresponding author on reasonable request.

## Abbreviations

BSL-4: Biosafety level 4
CFR: Case fatality rate
HCW: Health care worker
ICU: Intensive care unit
IPC: Infection prevention and control
LMIC: Low and middle-income country
MHFW: Ministry of Health and Family Welfare
NAIC: National Airborne Infection Control
NiV: Nipah virus
NiV-B: Nipah virus, Bangladesh subtype
NiV-M: Nipah virus, Malaysia subtype
OR: Odds ratio
PHEIC: Public health emergency of international concern
PICO: Population, intervention, comparison and outcome
PPE: Personal protective equipment
R&D: Research and development
SARS-CoV-2: Severe acute respiratory syndrome coronavirus 2
WHO: World Health Organization
