# Supplementary material for: The role of infection prevention and control in the mitigation of human-to-human transmission of Nipah virus: a systematic review
Source: Antimicrob Resist Infect Control. 2025 Nov 29;15:1. doi: 10.1186/s13756-025-01677-5 (PMC12771775; doi:10.1186/s13756-025-01677-5)
Supplement: Supplementary file 1 — Supplementary Material 1 [file 13756_2025_1677_MOESM1_ESM.docx]

**Supplementary File A: Literature search protocol**

Question 1: What are the modes of human-to-human transmission of NiV?

This question intends to supplement findings on effectiveness of IPC measures to prevent NiV infection.

Inclusion Criteria:

- Study design: Case-control studies, cohort studies and non-comparative studies. Case reports and case series are also included.
- Population: Individuals with confirmed or probable cases of NiV infection, as defined by the WHO's case definition for NiV disease or other local or regional guidelines. Suspected cases with a documented epidemiological link to a confirmed or probable case may also be considered.
- Outcomes: confirmed, probable or suspected NiV infection following exposure to a confirmed or probable case of NiV.

Each transmission reported as either a single mode of transmission, multiple possible routes of transmission, or unknown route of transmission. The following modes will be considered:

- Respiratory transmission (whether droplet or airborne)
- Direct contact with NiV case
- Indirect contact with NiV case, including fomite transmission
- Vertical (mother-to-child) transmission
- Sexual transmission
- Non-human related (zoonotic transmission)

Settings: all geographies are considered but given NiV outbreaks have only occurred in 5 countries (Bangladesh, India, Malaysia, Philippines, Singapore) it is expected that data will be obtained from these countries only.

Exclusion Criteria:

- Studies published in a language other than English.
- Studies that do not provide sufficient information to infer modes of transmission of NiV.
- Studies that refer exclusively to zoonotic transmission or spillover of NiV.

Search terms:

1. Nipah virus or Nipah.mp
2. Case*.mp
3. Person.mp
4. Individual.mp
5. 2 or 3 or 4
6. Outbreak.mp
7. Transmi*.mp
8. Spread.mp
9. Epidemic.mp
10. Cluster.mp
11. 6 or 7 or 8 or 9 or 10
12. Bangladesh.mp
13. India.mp
14. Malaysia.mp
15. Philippines.mp
16. Singapore.mp
17. 12 or 13 or 14 or 15 or 16
18. 1 and 5 and 11 and 17

Question 2: Which infection prevention and control precautions are most effective in preventing transmission of NiV to healthcare workers?

Inclusion Criteria:

- Study design: Comparative studies, including but not limited to randomised or otherwise controlled trials, cohort studies, case-control studies and ecological studies. In the event of no comparative studies evaluating the effectiveness of infection prevention and control measures in preventing NiV transmission being available, studies describing the use of IPC measures within NiV outbreaks will be included to inform, albeit not fully answer, this question.
- Population: Healthcare workers, patients and other populations within healthcare settings exposed to cases of NiV disease.
- Comparisons:
  - Intervention 1: isolation of Nipah patients in 'airborne precaution' rooms, defined as having a high ventilation rate and controlled direction of airflow.
  - Control 1: isolation of Nipah patients in 'adequately ventilated' rooms that lack a controlled direction of airflow, or rooms with insufficient ventilation to qualify as an airborne precaution room.
  - Intervention 2: use of respirators (e.g. N95, FFP2) during management of Nipah patients.
  - Control 2: use of medical masks during management of Nipah patients.
  - Intervention 3: use of alcohol-based hand hygiene among healthcare workers involved in management of Nipah patients.
  - Control 3: use of other, or lack of, hand hygiene among healthcare workers involved in management of Nipah patients.
- Outcomes: Nipah virus infection in healthcare workers, patients, and other populations within healthcare settings.

Settings: all geographies are considered, but given Nipah outbreaks have only occurred in 5 countries (Bangladesh, India, Malaysia, Philippines, Singapore) it is expected that data will be obtained from these countries only.

Exclusion Criteria:

- Studies published in a language other than English.
- Studies that do not refer to human Nipah cases.
- Studies that make no reference to evaluation of infection prevention and control practices.
- Non-comparative studies.

Search terms:

1. Nipah.mp
2. (random* or factorial* or placebo* or assign* or allocat* or crossover*).mp
3. ((blind* or mask*) and (single or double or triple or treble)).mp
4. crossover procedure.mp
5. double blind procedure or single blind procedure.mp
6. randomization/ or placebo.mp
7. randomized controlled trial.mp
8. cohort study.mp. or cohort analysis.mp
9. comparative study.mp
10. controlled study.mp
11. editorial or commentary.mp
12. 2 or 3 or 4 or 5 or 6 or 7
13. 8 or 9 or 10 NOT 11
14. 12 or 13
15. 1 and 14

Question 3: What are the risk factors for transmission of NiV within household or community settings?

Inclusion Criteria:

- Study design: Comparative studies, including but not limited to randomised or otherwise controlled trials, cohort studies, case-control studies and ecological studies, will initially be considered. Non-comparative studies will be included to add to findings from comparative studies.
- Population: Individuals with confirmed or probable cases of Nipah virus infection, as defined by the WHO's case definition for Nipah virus disease or other local or regional guidelines. Suspected cases with a documented epidemiological link to a confirmed or probable case may also be considered.
- Comparisons:
  - Intervention 1: Nipah patients isolate at home, with no contact with other household members between symptomatic onset and the end of the infectious period.
  - Control 1: Nipah patients do not isolate from family members during the infectious period.
- Outcomes: confirmed, probable or suspected Nipah virus infection following exposure to a confirmed or probable case of Nipah.

Settings: all geographies are considered, but given Nipah outbreaks have only occurred in 5 countries (Bangladesh, India, Malaysia, Philippines, Singapore) it is expected that data will be obtained from these countries only.

Exclusion Criteria:

- Studies published in a language other than English.
- Studies that do not refer to Nipah cases.
- Studies that do not document the occurrence or transmission of Nipah within a household setting.

Search terms:

1. Nipah virus or Nipah.mp
2. Case*.mp
3. Person.mp
4. Individual.mp
5. 2 or 3 or 4
6. Outbreak.mp
7. Transmi*.mp
8. Spread.mp
9. Epidemic.mp
10. Cluster.mp
11. 6 or 7 or 8 or 9 or 10
12. Bangladesh.mp
13. India.mp
14. Malaysia.mp
15. Philippines.mp
16. Singapore.mp
17. 12 or 13 or 14 or 15 or 16
18. 1 and 5 and 11 and 17

**Supplementary File B: Quality and risk of bias assessment results**

Case-control and cohort studies were assessed for quality and risk of bias by the following 13-question checklist, adapted from JBI Critical Appraisal tools for case-control and cohort studies:

| - 1. Were the groups comparable other than the presence/absence of exposure or outcome? |
| --- |
| - 1. Were cases and controls matched appropriately? |
| - 1. Were the same criteria used for identification of cases and controls/exposed and unexposed? |
| - 1. Was exposure measured in a standard, valid and reliable way? |
| - 1. Was exposure measured in the same way for cases and controls? |
| - 1. Were confounding factors identified? |
| - 1. Were strategies to deal with confounding factors stated? |
| - 1. Were outcomes assessed in a standard, valid and reliable way for each group? |
| - 1. Was the exposure period of interest long enough to be meaningful? |
| - 1. Was appropriate statistical analysis used? |
| - 1. Was the follow up time reported and sufficient to be long enough for outcomes to occur? |
| - 1. Was follow up complete, and if not, were the reasons to loss to follow up described and explored? |
| - 1. Were strategies to address incomplete follow up utilized? |

The results for each comparative study are presented below:

|  | Mounts et al 2001 | Smither et al 2022 | Parashar et al 2000 | Homaira et al 2010 | Huang et al 2022 | Chakraborty et al 2015 | Sazzad et al 2013 | Luby et al 2006 | Fogarty et al 2008 | Hegde et al 2016 | Hsu et al 2014 | Homaira et al 2010 | Gurley et al 2007 | Mont-gomery et al 2008 | Chew et al 2000 | Amal et al 2000 |
| --- | --- | --- | --- | --- | --- | --- | --- | --- | --- | --- | --- | --- | --- | --- | --- | --- |
| 1 | Yes | Yes | Yes | Yes | Yes | Yes | Yes | Yes | n/a | Yes | Mixed | Yes | Yes | Yes | Yes | No |
| 2 | n/a | n/a | Yes | Yes | n/a | Yes | Yes | Yes | n/a | Yes | No | Yes | Yes | Yes | Mixed | No |
| 3 | Yes | n/a | No | Yes | n/a | Yes | Yes | Yes | n/a | Yes | Yes | Yes | Yes | Yes | Yes | Yes |
| 4 | Yes | Yes | Yes | Yes | Yes | Yes | Yes | Yes | Yes | Yes | Yes | Yes | Yes | Yes | Yes | Yes |
| 5 | n/a | n/a | Yes | Yes | Yes | Yes | Yes | Yes | Yes | Yes | Yes | Yes | Yes | Yes | Yes | Yes |
| 6 | No | n/a | Yes | No | n/a | No | No | No | n/a | No | No | No | No | No | No | No |
| 7 | Yes | n/a | Yes | Yes | n/a | No | No | No | n/a | Yes | No | No | No | No | No | No |
| 8 | No | Yes | Yes | Mixed | Yes | Yes | Yes | Yes | Yes | Yes | Yes | Yes | Yes | Yes | Yes | Yes |
| 9 | Yes | Yes | Yes | Yes | Yes | Yes | Yes | Yes | Yes | Yes | Yes | Yes | Yes | Yes | Yes | Yes |
| 10 | Yes | Yes | Yes | Yes | Yes | Yes | Yes | Yes | Yes | Yes | Yes | Yes | Yes | Yes | Yes | Yes |
| 11 | Yes | Yes | n/a | n/a | Yes | Yes | n/a | n/a | n/a | n/a | n/a | n/a | n/a | n/a | n/a | n/a |
| 12 | Mixed | Yes | n/a | n/a | n/a | n/a | n/a | n/a | n/a | n/a | n/a | n/a | n/a | n/a | n/a | n/a |
| 13 | No | n/a | n/a | n/a | n/a | n/a | n/a | n/a | n/a | n/a | n/a | n/a | n/a | n/a | n/a | n/a |

Case series and retrospective case analyses were assessed via the JBI case series checklist:

| - - 1. Were there clear criteria for inclusion in the case series? |
| --- |
| - - 1. Was the condition measured in a standard, reliable way for all participants included in the case series? |
| - - 1. Were valid methods used for identification of the condition for all participants included in the case series? |
| - - 1. Did the case series have consecutive inclusion of participants? |
| - - 1. Did the case series have complete inclusion of participants? |
| - - 1. Was there clear reporting of the demographics of the participants in the study? |
| - - 1. Was there clear reporting of clinical information of the participants? |
| - - 1. Were the outcomes or follow up results of cases clearly reported? |
| - - 1. Was there clear reporting of the presenting site(s)/clinic(s) demographic information? |
| - - 1. Was statistical analysis appropriate? |

The results for each case series/retrospective case analysis are presented below:

|  | Lee et al 2020 | Chandni et al 2020 | Kumar et al 2024 | Ali et al 2020 | Chadha et al 2006 | Hassan et al 2018 | Thomas et al 2019 | Arunkumar et al 2019 | Yadav et al 2022 | Nikolay et al 2019 |
| --- | --- | --- | --- | --- | --- | --- | --- | --- | --- | --- |
| 1 | Yes | Yes | Yes | Yes | Yes | Yes | Yes | Yes | Yes | Yes |
| 2 | Unclear | Yes | Yes | Yes | Yes | Yes | Yes | Yes | Yes | Yes |
| 3 | Yes | Yes | Yes | Yes | Yes | Yes | Yes | Yes | Yes | Yes |
| 4 | No | No | No | No | No | No | No | No | No | No |
| 5 | Yes | Yes | Yes | Yes | Unclear | Yes | Yes | Yes | Yes | Yes |
| 6 | Yes | Yes | Yes | Yes | Yes | No | Yes | Yes | Yes | Yes |
| 7 | Yes | Yes | Yes | Yes | Yes | No | Yes | Yes | Yes | Yes |
| 8 | Yes | Yes | Yes | Yes | Yes | Yes | Mixed | Yes | Yes | Yes |
| 9 | No | No | No | No | No | No | No | No | No | No |
| 10 | Yes | Yes | Yes | No | Yes | Yes | Yes | No | n/a | Yes |

Case series were assessed via the corresponding JBI checklist:

| - 1. Were the criteria for inclusion in the sample clearly defined? |
| --- |
| - 1. Were the study subjects and the setting described in detail? |
| - 1. Was the exposure measured in a valid and reliable way? |
| - 1. Were objective, standard criteria used for measurement of the condition? |
| - 1. Were confounding factors identified? |
| - 1. Were strategies to deal with confounding factors stated? |
| - 1. Were the outcomes measured in a valid and reliable way? |
| - 1. Was appropriate statistical analysis used? |

The results for each cross-sectional study are presented below:

|  | Raj et al 2019 | Chan et al 2002 | Kumar et al 2020 | Luby et al 2009 | Gurley et al 2007 |
| --- | --- | --- | --- | --- | --- |
| 1 | Yes | Yes | Yes | Yes | Yes |
| 2 | Yes | Yes | Yes | Yes | Yes |
| 3 | Yes | Yes | Yes | Yes | Yes |
| 4 | Mixed | Yes | Yes | Yes | Yes |
| 5 | No | No | No | No | No |
| 6 | No | No | No | No | No |
| 7 | Yes | Yes | Yes | Yes | Yes |
| 8 | Yes | Yes | Yes | Yes | No |

**Supplementary File C: Overview of studies included for data extraction**

| **Study title** | **Year of publication** | **Country of study** | **Type of study** | **Stages of review (1-4) addressed** |
| --- | --- | --- | --- | --- |
| A cohort study of health care workers to assess nosocomial transmissibility of Nipah virus, Malaysia, 1999 | 2001 | Malaysia | Retrospective cohort | 2,3 |
| Assessment of Health Facilities for Airborne Infection Control Practices and Adherence to National Airborne Infection Control Guidelines: A Study from Kerala, Southern India. | 2019 | India | Cross-sectional | 2 |
| A survey of Nipah virus infection among various risk groups in Singapore | 2002 | Singapore | Cross-sectional | 3 |
| Case-control study of risk factors for human infection with a new zoonotic paramyxovirus, Nipah virus, during a 1998-1999 outbreak of severe encephalitis in Malaysia | 2000 | Malaysia | Case-control | 3 |
| Changing contact patterns over disease progression: Nipah virus as a case study | 2020 | Bangladesh | Cross-sectional | 1 |
| Clinical manifestations of nipah virus-infected patients who presented to the emergency department during an outbreak in Kerala State in India, May 2018 | 2020 | India | Case series | 1 |
| Clinico-epidemiological presentations and management of Nipah virus infection during the outbreak in Kozhikode district, Kerala state, India 2023 | 2024 | India | Case series | 1 |
| Cluster of nipah virus infection, kushtia district, Bangladesh, 2007 | 2010 | Bangladesh | Case-control | 1,3 |
| Dissecting an outbreak: A clinico-epidemiological study of Nipah virus infection in Kerala, India, 2018 | 2020 | India | Case series | 1 |
| Evolving epidemiology of Nipah virus infection in Bangladesh: Evidence from outbreaks during 2010-2011 | 2016 | Bangladesh | Case-control | 3 |
| Nipah Virus Infection Outbreak with Nosocomial and Corpse-to-Human Transmission, Bangladesh | 2013 | Bangladesh | Case-control | 1,3 |
| Foodborne transmission of Nipah virus, Bangladesh | 2006 | Bangladesh | Case-control | 3 |
| Infections among contacts of patients with Nipah virus, India | 2020 | India | Cross-sectional | 3 |
| Investigating Rare Risk Factors for Nipah Virus in Bangladesh: 2001-2012 | 2016 | Bangladesh | Case-control | 3 |
| Nipah virus-associated encephalitis outbreak, Siliguri, India | 2006 | India | Retrospective analysis | 1 |
| Nipah virus encephalitis reemergence, Bangladesh | 2004 | Bangladesh | Case-control | 1,3 |
| Nipah Virus Infection in Kozhikode, Kerala, South India, in 2018: Epidemiology of an Outbreak of an Emerging Disease. | 2019 | India | Case series | 1 |
| Nipah virus outbreak with person-to-person transmission in a district of Bangladesh, 2007 | 2010 | Bangladesh | Case-control | 1,3 |
| Person-to-person transmission of Nipah virus in a Bangladeshi community | 2007 | Bangladesh | Case-control | 1,3 |
| Outbreak investigation of nipah virus disease in Kerala, India, 2018 | 2019 | India | Case series | 1 |
| Recurrent zoonotic transmission of Nipah virus into humans, Bangladesh, 2001-2007 | 2009 | Bangladesh | Retrospective analysis | 1 |
| Risk factors for Nipah virus encephalitis in Bangladesh | 2008 | Bangladesh | Case-control | 1,3 |
| Risk factors for Nipah virus infection among abattoir workers in Singapore | 2000 | Singapore | Case-control | 3 |
| Risk factors for Nipah virus transmission, Port Dickson, Negeri Sembilan, Malaysia: results from a hospital-based case-control study | 2000 | Malaysia | Case-control | 3 |
| Risk of nosocomial transmission of nipah virus in a Bangladesh Hospital | 2007 | Bangladesh | Cross-sectional | 1,2 |
| Tackling a global epidemic threat: Nipah surveillance in Bangladesh, 2006-2021 | 2023 | Bangladesh | Retrospective analysis | 1 |
| Transmission of nipah virus - 14 years of investigations in Bangladesh | 2019 | Bangladesh | Retrospective analysis | 1 |
| Aerosol Survival, Disinfection and Formalin Inactivation of Nipah Virus | 2022 | United Kingdom | Experimental | 4 |
| Evaluation and comparison of three virucidal agents on inactivation of Nipah virus | 2022 | China | Experimental | 4 |
| Henipavirus susceptibility to environmental variables | 2007 | Australia | Experimental | 4 |
| Nipah virus contamination of hospital surfaces during outbreaks, Bangladesh, 2013-2014 | 2018 | Bangladesh | Retrospective analysis | 2 |
| Update: Outbreak of Nipah Virus – Malaysia and Singapore, 1999 | 1999 | Malaysia, Singapore | Outbreak report | 1 |

**Supplementary File D: Line list of NiV cases identified from the descriptive literature**

**Supplementary File E: Collection of odds ratios for meta-analysis of studies outlining contact with a NiV case as a risk factor for NiV transmission**
